# Australian chiropractors’ perception of the clinical relevance of anatomical sciences and adequacy of teaching in chiropractic curricula

**DOI:** 10.1186/s12998-020-00325-6

**Published:** 2020-07-16

**Authors:** Rosemary Giuriato, Goran Štrkalj, Tania Prvan, Nalini Pather

**Affiliations:** 1 Department of Anatomy, School of Medical Sciences, Faculty of Medicine, The University of New South Wales, Sydney, New South Wales Australia; 2 Department of Chiropractic, Faculty of Science and Engineering, Macquarie University, Macquarie Park, Australia; 3 Department of Mathematics and Statistics, Faculty of Science and Engineering, Macquarie University, Sydney, New South Wales Australia

**Keywords:** Anatomy, Chiropractic, Education

## Abstract

**Background:**

Human anatomy education is compulsory in the undergraduate curriculum in all Australian chiropractic education programs. There is very little data on clinicians’ perceptions of the adequacy of their anatomy training and its relevance to practice. The aims of this study were to evaluate Australian registered chiropractors’ perceptions on the relevance and adequacy of anatomy training for clinical practice and analyse their opinion on the usefulness of the teaching resources utilized during their preprofessional training.

**Methods:**

A questionnaire-based survey was conducted on a sample of Australian registered chiropractors focussing on the adequacy of their anatomical science (gross anatomy, histology, neuroanatomy and embryology) training and the clinical relevance of each individual sub-discipline, and the perceived value of each of the different anatomy teaching resources utilized.

**Results:**

A total of 128 completed surveys were returned from an estimated 387 attendees at two national chiropractic conferences (estimated 33% response rate). The respondents represent 2.6% of registered chiropractors in Australia in 2016 and were representative in terms of gender (66.4% male) but not age, with older generations being over-represented (peak age group 35–44 vs. 25–34). The majority of the survey respondents obtained their chiropractic qualification in Australia (89.1%) and graduated after 1990 with an average of 21.7 years (SD = 11.3, range = 1–44) in practice. Respondents were equally likely to have undertaken anatomy training in Medicine, Science, Health Science, or other faculties. The disciplines perceived most relevant for clinical practice were neuroanatomy (100% of respondents agreeing) and gross anatomy (99.2%), followed by histology (86.0%) and embryology (81.1%). Respondents also perceived their training to be most adequate in neuroanatomy (99.3%) and gross anatomy (99.2%) followed by histology (91.4%) and embryology (85%). Respondents confirmed exposure to a varied suite of anatomy teaching tools utilized during their pre-professional training and highly valued access to cadavers and prosected specimens.

**Conclusions:**

The majority of respondents perceived anatomy as highly relevant to their clinical practice and noted that it was adequately taught within a wide range of educational approaches. These results will assist educators to refine content and delivery of anatomy course offerings to maximize relevance in chiropractic clinical practice.

## Background

Chiropractors are allied health professionals with a focus on “the diagnosis, treatment and prevention of mechanical disorders of the musculoskeletal system” [1]. Chiropractors apply a range of manual therapeutic techniques directed to the patient’s spine and extremities with the aim to restore joint function, reduce pain and encourage mobility [2]. Given that musculoskeletal conditions are highly prevalent [3] and that approximately one in three Australians seek chiropractic care in a 12 month period [4], it is important to ascertain whether Australian chiropractors perceive their training in anatomy adequate to provide competent diagnoses and management of musculoskeletal conditions. As primary healthcare providers, patients are able to access chiropractic services without the need of a health practitioner’s referral. Sound anatomical knowledge is essential for the safe delivery of manipulative techniques required as a part of chiropractic clinical practice [5].

In Australia, evidence-based chiropractic education offered in public universities can be traced back to 1990 when the Sydney College of Chiropractic merged with Macquarie University in Sydney, to provide the first chiropractic program at a government funded public university in the world [6, 7]. Registration to practice chiropractic requires the completion of an undergraduate and/or postgraduate degree from one of four accredited programs in Australia [8]. Regulation of chiropractic education in Australia has been the responsibility of the Council on Chiropractic Education Australasia (CCEA) since 2010, when the profession was included in the National Registration and Accreditation Scheme. The CCEA formulates and oversees the accreditation standards applicable to chiropractic programs [9]. Previously published educational standards for the first professional chiropractic award programs (2009–2017) [10] identified the need to incorporate biomedical sciences in the chiropractic program curriculum. These biomedical sciences included “anatomy, biochemistry, physiology, neurology, microbiology, histology, embryology, pathology, biophysics, molecular and cell biology, genetics, immunology, and other appropriate subjects” [10]. These standards did not prescribe core content but rather made the recommendation that the biomedical sciences were to “ensure an in-depth understanding of basic biological principles, consisting of a core of information on the fundamental structures, functions and interrelationships of the body systems” leading to the “understanding of the scientific knowledge, concepts and methods fundamental to acquiring and applying clinical science” [10].

Currently, all four chiropractic programs delivered in Australia include human anatomy courses as a compulsory requirement in their undergraduate curriculums [8, 11]. Australian chiropractic programs follow a sequential training program, progressing the student from the foundational biomedical sciences in the undergraduate years to full clinical internship or work integrated units at the end of their academic programs.

Relevance of education to clinical practice is one of the most important aspects of teaching and learning [12]. To date, there exists little information on chiropractors’ perceived relevance and adequacy of their anatomy education to their clinical practice. Previous research exploring pre-professional anatomy education within chiropractic programs confirmed considerable investment in anatomy teaching resources [8, 11, 13] but provided little insight into chiropractors’ perceptions of the usefulness of these teaching tools. Determining chiropractors’ perceptions of these tools will assist to develop anatomy curricula appropriate for chiropractic clinical practice. Clinicians are uniquely able to offer input on anatomical sciences education as they are at the forefront of both the theoretical application of knowledge to clinical practice and of changes to the practice of the profession [14, 15].

Analysis of chiropractors’ opinion on the application of knowledge adopted during their education to clinical practice is imperative for investigating the relevance and adequacy of their education and informs educators on possible changes that may be considered in future course offerings. It also provides an opportunity to reflect on adequacy of curriculum content and usefulness for clinical application.

### Aims

The aims of this study were to evaluate Australian registered chiropractors’ perceptions of the relevance and adequacy of anatomy training for clinical practice and determine their opinion on the usefulness of the teaching resources utilized during their anatomy training.

## Methods

This study used a questionnaire-based survey tool modified from similar previously published surveys which evaluated the relevance of gross human anatomy in clinical medical practice [16, 17] and the methods used to study anatomy [18]. The survey was modified to suit chiropractic clinicians and refined in consultation with four academic experts in anatomy and four chiropractic clinicians, prior to distribution.

The questionnaire collected demographic data (gender, age), year of graduation, years in clinical practice, country in which degree was completed, institution conferring qualification (for extrapolation of country data), home faculty delivering anatomy content, qualifications of anatomy staff and the usefulness of the anatomy teaching resources used during their training. The questionnaire also posed questions regarding the relevance of anatomy, adequacy of anatomical training, rated on a 6-point Likert scale (strongly disagree, disagree, somewhat disagree, somewhat agree, agree, strongly agree), and value of various education resources, rated on a 7-point Likert-type scale (extremely helpful, quite helpful, moderately helpful, slightly helpful, not at all helpful, not sure, or was not used) [19].

The surveys were anonymously completed. Printed copies of the survey were distributed at the start of the two Australian chiropractic association’s national conferences held in Sydney in late 2016 (Chiropractic Australia) and in Canberra in early 2017 (Australian Chiropractors Association). Ethical approval was obtained from the Human Research Ethics Committee of UNSW, Sydney (HC17058).

### Data analysis

Descriptive statistics were used to compare respondents’ age and gender distribution to registered chiropractors in Australia (2016) and to test the homogeneity in the distribution of country where qualification was obtained (Australia, elsewhere), year of chiropractic qualification (< 1978 1981–1990, > 1990), and anatomy home-faculty (Medicine, Science, Health Science, other). Cut points for year of chiropractic qualification were selected based on key periods for the Chiropractic profession in Australian history (1978: registration of chiropractic as a profession; 1990: first public university-based education program). The age of respondents was grouped in 10-year intervals (< 25, 25–34, 35–44, 45–54, 55–64, > 65 years) and the distribution compared against the population of Australian registered chiropractors in 2016 [20].

The Chi-squared test was used to test the homogeneity in distribution of responses for adequacy in training and clinical relevance for each of the sub-disciplines (gross anatomy, histology, neuroanatomy, embryology). Response categories were collapsed [21] and grouped by negative responses (strongly disagree, disagree, somewhat disagree), or positive responses (somewhat agree, agree, strongly agree). Statistical analysis was completed using SPSS Statistics for Windows, version 23.0.0.0 (IBM Corp., Armonk, NY).

## Results

### Study population

Based on conference registrations, it is estimated that 387 chiropractors attended one or both of the two professional associations’ national conferences, of which 128 (33%) attendees completed the survey. Attendees were requested to only complete the survey once. This represented 2.6% of registered chiropractors in Australia in 2016. The majority of survey respondents were male (66.4%) representing a gender distribution representative of registered chiropractors in Australia in 2016 (61.9% male, *p =* 0.29) (Table 1). The sample of 128 chiropractors had significantly different proportions in each age grouping compared to registered chiropractors in Australia (*p* < 0.001) with a greater proportion of respondents represented in older age groups.

**Table 1:** Survey respondent’s demographics compared to Australian registered chiropractors in 2016

	Respondents n (%)	Registered Chiropractors in 2016 n (%)
Gender^a^		
Male	85 (66.4)	3024 (61.9)
Female	43 (33.6)	1864 (38.1)
Total	128 (100)	4888 (100)
Age (years)		
< 25	2 (1.6)	105 (2.1)
25–34	15 (11.7)	1586 (32.4)
35–44	37 (28.9)	1422 (29.1)
45–54	34 (26.6)	941 (19.3)
55–64	34 (26.6)	570 (11.7)
> 65	6 (4.7)	264 (5.4)
Country of qualification^b^		
Australia	114 (89.1)	
Elsewhere	14 (10.9)	
Year of qualification^c^		
< 1980	15 (11.7)	
1981–99	33 (25.8)	
> 1990	80 (62.5)	
Anatomy home-faculty^d^		
Medicine	28 (21.9)	
Science	38 (29.7)	
Health science	33 (25.8)	
Other	21 (16.4)	
Unsure	8 (6.3)	

^a^Participants were provided with options of male, female, other

^b^The majority of respondents qualified in Australia (*p* < 0.001, Chi-square Test)

^c^Respondents were not evenly distributed across year of qualification (*p* < 0.001, Chi-square Test)

^d^Respondents were evenly distributed for anatomy home-faculty (excluding group: unsure) (*p* = 0.153, Chi-square Test)

The majority of the survey respondents obtained their chiropractic qualification in Australia (89.1%, *p* < 0.001) and qualified after 1990 *(p* < 0.001), with a mean of 21.7 (SD 11.3) years and range of 1 to 44 years in practice. Respondents were equally likely to be taught anatomy in Medicine, Science, Health Science, or another faculty (*p* = 0.153) (Table 1).

### Anatomy education: adequacy and relevance to clinical practice

The majority of respondents either agreed or strongly agreed that the anatomy taught was adequate across all sub-disciplines for clinical practice (Table 2). The highest levels of agreement were in the sub-disciplines of gross anatomy, neuroanatomy, and histology. A similar pattern of response was observed regarding relevance of anatomical training to practice as a chiropractor (Table 2). The sub-discipline of embryology returned the highest proportion of negative response for both adequacy and relevance to practice with proportions of 15.0 and 18.9% respectively.

**Table 2:** Proportion agreement that training in anatomy and knowledge in anatomy was adequate for practice

Anatomical sciences sub-discipline	Strongly disagree or disagree	Somewhat disagree	Somewhat agree	Agree or strongly agree	Comparison across these responses
	n (%)	n (%)	n (%)	n (%)	*p* value*
**Training in**					
Gross anatomy	0 (0)	1 (0.8)	6 (4.7)	121 (94.5)	< 0.001
Histology	5 (3.9)	6 (4.7)	26 (20.3)	91 (71.1)	< 0.001
Neuroanatomy	1 (0.8)	0 (0)	13 (10.2)	114 (89.1)	< 0.001
Embryology	9 (7.1)	10 (7.9)	29 (22.8)	79 (62.2)	< 0.001
**Knowledge in**					
Gross anatomy	0 (0)	1 (0.8)	1 (0.8)	126 (98.4)	< 0.001
Histology	9 (7.1)	7 (5.6)	55 (43.0)	55 (43.0)	< 0.001
Neuroanatomy	11 (8.7)	13 (10.2)	38 (29.9)	65 (51.2)	< 0.001
Embryology	11 (8.7)	13 (10.2)	38 (29.9)	65 (51.2)	< 0.001

*Chi-squared test used to test if proportions are the same across SD/D, Somewhat D, Somewhat A, A/SA responses

### Proportion of anatomy educators with a chiropractic qualification

Regarding the qualifications of the anatomy educators of the respondents; only 39 out of 128 (30.5%) were taught by academics with chiropractic qualifications, while 41 (32.0%) were taught by academics with some clinical qualifications, such as medical or other allied health, and 41 (32%) were taught by academics with no clinical qualifications. Seven (5.5%) respondents were not aware of the qualifications of academic staff.

### Utility of different teaching tools

Respondents had diverse perceptions of the usefulness of anatomy teaching tools used in their training (Table 3). The teaching tool rated most useful by respondents was cadaveric dissection followed by prosected cadavers, while the tool perceived as least useful was body painting surface anatomy. A large proportion of respondents were either unsure or had not used digital imaging (44.5%) or interactive anatomy software (61.7%) during their anatomy education.

**Table 3:** Respondents’ perceptions of the utility of anatomy teaching tools and strategies

Teaching resource	Number	Not helpful	Slightly or moderately helpful	Quite or extremely helpful	Not used or unsure of utility
	(n)	n (%)^a^	n (%)^a^	n (%)^a^	n (%)^b^
Cadaveric dissection	102	1 (1.0)	10 (9.8)	91 (89.2)	26 (20.3)
Prosected human cadavers	110	0 (0)	12 (10.9)	98 (89.1)	15 (11.7)
Didactic lectures	115	0 (0)	17 (14.8)	98 (85.2)	7 (5.5)
Medical imaging (e.g. X-ray)	122	0 (0)	24 (19.7)	98 (80.3)	6 (4.7)
Interactive collaborative	106	1 (0.9)	20 (18.9)	85 (80.2)	21 (16.4)
Reference text	125	0 (0)	26 (20.8)	99 (79.2)	2 (1.6)
Digital imaging (atlas, 2-D, 3-D)	70	0 (0)	17 (24.3)	53 (75.7)	57 (44.5)
Plastinated specimens	98	1 (1.0)	30 (30.6)	67 (68.4)	28 (21.9)
Models	122	1 (0.8)	40 (32.8)	81 (66.4)	5 (3.9)
Interactive anatomy software^a^	45	0 (0)	15 (33.3)	30 (66.7)	79 (61.7)
Laboratory manuals	111	1 (0.9)	41 (36.9)	69 (62.2)	16 (12.5)
Body painting surface anatomy	55	2 (3.6)	26 (47.3)	27 (49.1)	72 (56.3)

^a^Proportion of respondents with an opinion on helpfulness expressed as a percentage

^b^Proportion of respondents who answered question who had either not used the utility or were unsure if they had used it expressed as a percentage

## Discussion

This survey is the first to investigate the perception of Australian registered chiropractors on the relevance and adequacy of their anatomy education to their clinical practice. The study found that the majority of respondents agreed that their professional degree training provided them with adequate anatomy knowledge for clinical practice. Respondents perceived all of the four sub-disciplines of gross anatomy, neurology, histology, and embryology to be relevant to their daily clinical practice. The highest levels of agreement for both adequacy of training and relevance for clinical practice were in the sub-disciplines of gross anatomy, neuroanatomy, and histology. The respondents rated the most useful teaching resources to be cadaveric dissection followed by access to prosected cadavers. One possible reason for the general homogeneity in responses is that most of the respondents graduated from Australian training programs (89%) and that these educational programs are not significantly dissimilar [8].

The findings of this study are similar to the opinions of medical doctors and other allied health professionals who also considered anatomy to be highly relevant to their daily clinical practice [16, 17, 22, 23]. Osteopaths very similarly rated gross anatomy and neurology as the most relevant subdisciplines to their clinical practice [24]. The similarity of findings is possibly due to interdisciplinary overlap [25].

The finding that the respondents perceived their training to be relatively less adequate in the sub-disciplines of embryology and histology could be linked to their perception of the same subdisciplines as least relevant to their clinical practice. This may be reflective of the situation that embryology and histology were not always taught in all Australian chiropractic programs and if taught, possibly not directly linked to professional practice [8]. The rationale for the exclusion of these subdisciplines in chiropractic curriculum was beyond the scope of this survey however, it does raise questions regarding the relevance and core content of the disciplines that are considered a requirement for chiropractic clinical practice. There is no literature available in this area. The consequences of not including embryology and histology in chiropractic training may potentially impact professional practice, particularly in relation to the clinical understanding of a wide range of musculoskeletal pathologies. This has the potential to negatively impact the profession’s role in patient education and professional status. Furthermore, the knowledge gap could adversely impact future interdisciplinary collaboration for the chiropractic profession and threaten its position at the nexus between mainstream and alternative medicine [26].

### Range of teaching resources

Anatomy is a discipline that utilizes a wide range of teaching tools and strategies. The survey confirmed that chiropractors were exposed to a suite of teaching tools and strategies rather than a single resource (Table 3), similar to that utilized in medical anatomy teaching [27]. The use of a varied range of teaching tools indicates that anatomy was taught in an enriched format including formal, informal, didactic (traditional) and student-directed pedagogies. It also confirms that anatomy teachers were cognizant of the usefulness of each of the tools and implemented these in ways that enabled learning amongst the diverse student cohorts over time [28, 29].

The majority of respondents in this survey (62.5%) were taught anatomy by at least one anatomy academic with a clinical qualification, with one-third of respondents indicating that all of the staff involved in their anatomy education held chiropractic qualifications. This is not an accreditation criterion stipulated by the Council on Chiropractic Education Australasia (CCEA) but remains at the discretion of the teaching program [9]. The response is suggestive of the value that is placed on clinicians’ input (e.g. through demonstration of clinical relevance) into the teaching programs [30] and may reflect both the availability of clinicians who can teach anatomy and the acknowledgement of the importance of clinical competency by the course coordinators. The benefit of correlating anatomical content to clinical relevance also assists to mentor students towards possible future academic career paths, in addition to ensuring that anatomy is taught and adapted to ‘real-world’ clinical scenarios [31, 32].

Irrespective of whether chiropractic anatomists deliver the anatomy content, anatomy is often further integrated with content delivered subsequently within chiropractic education programs in a spiral program design. For some of the later content such as chiropractic manipulative technique, anatomy is both foundational and imperatively provides an opportunity to reinforce clinically relevant anatomy [33]. This symbiotic relationship is further enhanced when relevant chiropractic content is taught in parallel with anatomy, thus aligning anatomical theory with clinical decision making [30, 34, 35]. This is equally as useful in the undergraduate years when the students are learning anatomy for the first time as it is in the later years of their programs.

### The utility of teaching resources

The survey respondents highly regarded cadaveric learning as a teaching tool with the majority of respondents reporting access to human cadavers either via dissection (80%) or prosected specimens (86%) during their training, and they were perceived as the most helpful of all the teaching tools. This is in line with the argument made by many anatomy and medical educators that cadavers, whether via dissection or examination of prosected body parts, present an invaluable educational resource [28]. Access to human cadavers as a teaching tool provides an opportunity for students to develop three-dimensional perspectives of the human body and to appreciate clinically relevant anatomical variations [36–38]. This enhanced spatial awareness may assist with integrating anatomy knowledge to the application of clinical techniques [39]. The chiropractic profession’s predilection towards a hands-on approach may also be a reason why the respondents perceived cadaveric dissection and use of prosected specimens as the most helpful of the anatomy teaching tools [40]. The opportunity to learn from the experience of interacting with human cadavers is important to health education. For example, access to human cadavers serves to introduce students to the concept of death and instils respect and empathy from very early in their professional training [41].

In addition to the hands-on exposure to cadavers, the majority of respondents also agreed that the use of didactic lectures was a useful learning tool. This may be attributed to the efficiency by which didactic lectures are able to deliver a large volume of information and highlight the most relevant aspects of a body of knowledge [42]. Student perceptions of conventional didactic lectures are thought to be dependent on the lecturer delivering the material, which may have influenced responses for those who reported exposure to educators with clinical experience [43], who could demonstrate use of content knowledge in a clinical setting.

The use of radiological imaging was also perceived by the respondents to be valuable. The use of radiological images as a teaching resource serves to enhance students’ structural competency and provides a link between understanding the 2D representation to the anatomy in situ [44]. This finding may reflect that some chiropractors in Australia use spinal x-rays for assessing spine and intersegmental alignment to support clinical decision making. Chiropractors are eligible for licensure to use and own x-ray machines in addition to having referral rights to medical radiologists for spinal images [45].

In contrast, the teaching tools least utilized by the respondents included the use of interactive software, digital imaging 2D and 3D atlases along with the use of surface anatomy body painting, possibly reflective of the age of the respondents. As most of the respondents are of a relatively older generation, they may not have found the digital resources as useful as the current generation of learners, the so-called “digital natives” [46]. It should also be noted that these digital resources might not have been available in a sophisticated, easy to use form, as they are today, and therefore not deemed useful as compared to traditional anatomy teaching resources.

### Study limitations

The small sample size may not capture the full range of opinions and perceptions possible, which may limit the generalizability of the study findings. This study should be interpreted within the context of sample limitations and it should be noted that certain responses are based on respondents accurately remembering details, for example, the qualifications of their educators. The study was further limited in that it was not possible to determine the core content that participants received in each sub-discipline. This was beyond the scope of this survey. It is unclear if the level of exposure to certain teaching resources during training, has influenced the survey results. Most of the respondents were educated in Australia, therefore the findings may not be generalizable to other countries.

### Directions for future research

This study identified heterogeneity in the perceived relevance across anatomy subdisciplines, along with a wide variation in the utilization of teaching resources. It is unclear if the observed variation may reflect a lack of detailed guidance from the accrediting body on recommended course structure, or some other mechanism. Future studies may explore the drivers of individual program content, staffing and modes of delivery for anatomy education. In addition, it is unclear if the self-reported high-level agreement that training in anatomy was adequate, aligns with objective assessment of anatomical knowledge retained by chiropractors in the clinical setting. Future studies that seek to measure the anatomical knowledge of chiropractors should be conducted.

## Conclusion

Chiropractic is a primary contact clinical discipline relying on the application of anatomy knowledge for sound diagnosis and management of musculoskeletal health conditions. Most respondents agreed that training in gross anatomy and neuroanatomy was adequate and highly relevant to their practice. In contrast, whilst approximately two-thirds of respondents agreed that training in histology and embryology was adequate, they rated these anatomy subdisciplines as less relevant to clinical practice. The majority of respondents indicated that they were taught anatomy by clinically qualified staff, with access to a varied suite of teaching tools, of which cadaveric dissection and use of prosected specimens were perceived as the most beneficial to their clinical practice.

This study highlights the importance of including current clinicians’ opinions when reviewing anatomy courses in chiropractic degree programs. The results of this study may inform future delivery of anatomical content in academic programs for the chiropractic profession.

## Data Availability

The datasets analysed during the current study are held in the Research Data Management at UNSW repository: D0237583 Survey of health professionals’ perceptions of the appropriateness of the anatomy learnt during their professional training for practice and available on request.

## Abbreviations

CCEA: Council on Chiropractic Education Australasia
